# Supplementary material for: Delivery and Safety of a Two-Dose Preventive Ebola Virus Disease Vaccine in Pregnant and Non-Pregnant Participants during an Outbreak in the Democratic Republic of the Congo
Source: Vaccines (Basel). 2024 Jul 23;12(8):825. doi: 10.3390/vaccines12080825 (PMC11359453; doi:10.3390/vaccines12080825)
Supplement: Supplementary file 1 [file vaccines-12-00825-s001.zip › vaccines-3038591-supplementary.pdf]

Delivery and safety of a two-dose preventive Ebola disease vaccine in pregnant and non-pregnant participants during an outbreak in the Democratic Republic of the Congo

Supplementary materials

Table of Contents

1. SUPPLEMENTARY TABLES 2

Supplementary table S1. Timing of receipt of vaccination dose 2. 2

Supplementary table S2. Enrolment of subsets. 3

Supplementary table S3. Incidence of serious adverse events in individuals never reporting a pregnancy 4

Supplementary table S4: List of SAEs with fatal outcome 5

Supplementary table S5. Timing of exposure to each vaccine during pregnancy. 7

Supplementary table S6. Serious adverse events in women ever reporting a pregnancy: by exposure to each dose and overall 9

Supplementary table S7: Incidence of reasons given for caesarean section 10

Supplementary table S8: Pregnancy outcomes overall, by pregnancy subsets and by exposure during pregnancy 11

Supplementary Table S9: Congenital anomaly SAE reports 12

2. SUPPLEMENTARY INFORMATION 13

Supplementary Information S1: Statistical methods 13

References 14

1. SUPPLEMENTARY TABLES

Supplementary table S1. Timing of receipt of vaccination dose 2.

			**Non-pregnant individuals†**		**Pregnancy**	**Subsets**			
**Receipt of dose 2:**		Overall*	Adults ≥18 years old (all)	Children <18 years old (all)	Women ever pregnant during study	Adult subset	Child subset	Pregnant at dose 1 subset	Pregnant 30 days subset‡
Vaccinated with dose 1, N		20408	12596	6590	1221	494	492	272	88
Vaccinated with dose 2, n (%)		15328 (75.1)	9108 (72.3)	5232 (79.4)	988 (80.9)	420 (85)	449 (91.3)	233 (85.7)	81 (92.0)
Dose 2 *within window*: 56 (-14 +28) days of dose 1, n (%)		9280 (45.5)	6076 (48.2)	2732 (41.5)	472 (38.7)	368 (74.5)	397 (80.7)	200 (73.5)	40 (45.5)
Dose 2 *outside of window*: *early* (<42 days post dose 1), n (%)		5 (<0.1)	4 (<0.1)	1 (<0.1)	0 (0)	0 (0)	0 (0)	0 (0)	0 (0)
Dose 2 *outside of window*: *late* (>84 days post dose 1), n (%)		6043 (29.6)	3028 (24.0)	2499 (37.9)	516 (42.3)	52 (10.5)	52 (10.6)	33 (12.1)	41 (46.6)
Late receipt of dose 2, in months post dose 1§	4-6, n (%)	266 (1.3)	201 (1.6)	50 (0.8)	15 (1.2)	39 (7.9)	29 (5.9)	10 (3.7)	1 (1.1)
	7-8, n (%)	606 (3.0)	252 (2.0)	294 (4.5)	60 (4.9)	0 (0)	0 (0)	0 (0)	6 (6.8)
	9-10, n (%)	2519 (12.3)	1160 (9.2)	1128 (17.1)	231 (18.9)	0 (0)	0 (0)	1 (0.4)	20 (22.7)
	11-12, n (%)	1892 (9.3)	913 (7.2)	833 (12.6)	146 (12.0)	3 (0.6)	6 (1.2)	7 (2.6)	11 (12.5)
	13-15, n (%)	760 (3.7)	502 (4.0)	194 (2.9)	64 (5.2)	10 (2.0)	17 (3.5)	15 (5.5)	3 (3.4)
Did not receive dose 2 (died or contraindication), n (%)		10 (<0.1)	9 (0.1)	1 (<0.1)	0 (0)	0 (0)	0 (0)	0 (0)	0 (0)
Did not receive dose 2 (LTFU), n (%)		5070 (24.8)	3479 (27.6)	1357 (20.6)	233 (19.1)	74 (15.0)	43 (8.7)	0 (0)	0 (0)

*Overall N = non-pregnant individuals + ever pregnant (unless difference of 1 occurs, due to 1 participant with missing age and sex, possibly a male of working age who reported their profession as a mechanic).

†Non-pregnant individuals with a missing value for age: n=1.

‡Subset of women with conception up to 30 days post dose 1, or within 30 days either side of dose 2.

§ D84 = 3 months (84 days) post dose 1; denominator is number who received dose 1

Supplementary table S2. Enrolment of subsets.

**Number of participants vaccinated with dose 1 in each subset:**		
Adult safety subset* (≥18 years, excluding pregnant women), N		494
Child safety subset* (<18 years, excluding pregnant women), N		492
Women pregnant at dose 1 subset*, N		272
Trimester at dose 1†, n (%)	1: <13 weeks	60 (22.1)
	2: 13-26 weeks	69 (25.4)
	3: 27-43 weeks	91 (33.5)
	Unknown†	52 (19.1)
Women pregnant within 30 days of either dose 1 subset*, N		88
Category†, n (%)	Conception 1-30 days post dose 1	48 (54.5)
	Conception 1-30 days pre-dose 2	26 (29.5)
	Conception 1-30 days post dose 2	5 (5.7)
	Unconfirmed if before or after dose 2 (date LMP missing)	9 (10.2)
Infant examination		87

* The target size for each of the adult and child safety subsets was 500 and this was met; the reduction seen here is due to movement of female participants to the pregnancy subset upon notification of a pregnancy and exclusion of one adult and one child whose informed consent documents could not be found during archiving. The target size of the subset of women pregnant at dose 1 was 250; two women were excluded due to not being able to locate the informed consent forms during archiving and 24 additional women were inadvertently enrolled. The target size of the subset of women pregnant within 30 days of dose 1 or 2 was 250; 88 women provided consent to be enrolled in the subset.

† Gestational age in weeks at the time of vaccination is calculated from date of conception, based on the date of last menstrual period (LMP) where available. When the gestational age is unknown, it means that the date of last menstrual period is not available for that pregnancy. Date of conception was calculated as date of LMP + 14 days.

Supplementary table S3. Incidence of serious adverse events in individuals never reporting a pregnancy.

MedDRA Preferred Term by System Organ Class	Adults (≥18 years old)	Children (<18 years old)	All individuals never reporting a pregnancy
Number of participants vaccinated	12596	6590	19187
**Congenital, familial, and genetic disorders**			
Hydrocele	0 (0)	1 (<0.1)	1 (<0.1)
**Endocrine disorders**			
Diabetes mellitus inadequate control	1 (<0.1)	0 (0)	1 (<0.1)
**Gastrointestinal disorders**			
Abdominal strangulated hernia	1 (<0.1)	0 (0)	1 (<0.1)
Enteritis	1 (<0.1)	0 (0)	1 (<0.1)
Gastritis	3 (<0.1)	0 (0)	3 (<0.1)
Intestinal obstruction	0 (0)	1 (<0.1)	1 (<0.1)
Umbilical hernia	1 (<0.1)	0 (0)	1 (<0.1)
**General disorders and administration site conditions**			
Multiple organ dysfunction syndrome	1 (<0.1)	0 (0)	1 (<0.1)
**Hepatobiliary disorders**			
Hepatic cyst	1 (<0.1)	0 (0)	1 (<0.1)
**Immune system disorders**			
Anaphylactic shock	1 (<0.1)	0 (0)	1 (<0.1)
**Infections and infestations**			
Gastroenteritis	1 (<0.1)	2 (<0.1)	3 (<0.1)
Malaria	6 (<0.1)	3 (<0.1)	9 (<0.1)
Pyelonephritis	2 (<0.1)	0 (0)	2 (<0.1)
Sepsis	0 (0)	1 (<0.1)	1 (<0.1)
Skin bacterial infection	1 (<0.1)	0 (0)	1 (<0.1)
Typhoid fever	6 (<0.1)	0 (0)	6 (<0.1)
Upper respiratory tract infection	1 (<0.1)	0 (0)	1 (<0.1)
Urinary tract infection	3 (<0.1)	1 (<0.1)	4 (<0.1)
**Injury, poisoning and procedural complications**			
Craniocerebral injury	2 (<0.1)	0 (0)	2 (<0.1)
Femur fracture	1 (<0.1)	0 (0)	1 (<0.1)
Fibula fracture	1 (<0.1)	0 (0)	1 (<0.1)
Jaw fracture	1 (<0.1)	0 (0)	1 (<0.1)
Procedural shock	1 (<0.1)	0 (0)	1 (<0.1)
Road traffic accident	1 (<0.1)	1 (<0.1)	2 (<0.1)

MedDRA Preferred Term by System Organ Class	Adults (≥18 years old)	Children (<18 years old)	All individuals never reporting a pregnancy
Number of participants vaccinated	12596	6590	19187
Snake bite	2 (<0.1)	0 (0)	2 (<0.1)
Thermal burn	1 (<0.1)	0 (0)	1 (<0.1)
**Metabolism and nutrition disorders**			
Hypoglycaemia	1 (<0.1)	0 (0)	1 (<0.1)
Malnutrition	0 (0)	1 (<0.1)	1 (<0.1)
**Musculoskeletal and connective tissue disorders**			
Vertebral osteophyte	1 (<0.1)	0 (0)	1 (<0.1)
**Respiratory, thoracic, and mediastinal disorders**			
Asthma	1 (<0.1)	0 (0)	1 (<0.1)
Lower respiratory tract infection	0 (0)	1 (<0.1)	1 (<0.1)
Pneumonia	0 (0)	1 (<0.1)	1 (<0.1)
Pulmonary tuberculosis	1 (<0.1)	0 (0)	1 (<0.1)
**Skin and subcutaneous tissue disorders**			
Keloid scar	1 (<0.1)	0 (0)	1 (<0.1)
**Surgical and medical procedures**			
Appendicectomy	1 (<0.1)	0 (0)	1 (<0.1)
Knee operation	1 (<0.1)	0 (0)	1 (<0.1)
**Vascular disorders**			
Shock haemorrhagic	1 (<0.1)	0 (0)	1 (<0.1)

Data are n (%) of subjects with 1 or more events.

Supplementary table S4: List of SAEs with fatal outcome

					At the onset of SAE					
**Participant**	**SAE**	**Age (years)**	**Sex**	**MedDRA Preferred Term**	**Days since dose 1**	**Days since dose 2**	**Gestation (weeks) at end of pregnancy**	**Causality dose 1**	**Causality dose 2**	**Cause of death**
1	1	62	Male	Gastroenteritis	42	No dose 2	n/a	Not related		ACUTE NON-FEBRILE ENTERITIS WITH SEVERE DEHYDRATION & SHOCK

					At the onset of SAE					
**Participant**	**SAE**	**Age (years)**	**Sex**	**MedDRA Preferred Term**	**Days since dose 1**	**Days since dose 2**	**Gestation (weeks) at end of pregnancy**	**Causality dose 1**	**Causality dose 2**	**Cause of death**
	2			Multiple organ dysfunction syndrome	42	No dose 2	n/a	Not related		EDEMA-ASCITIC SYNDROME POSSIBLE HEPATIC INSUFFICIENCY DECOMPENSATED HEART DISEASE
2	1	19	Male	Thermal burn	35	8	n/a	Not related	Not related	DEATH THERMAL BURN
3	1	79	Female	Procedural shock	41	No dose 2	n/a	Not related		GASTRIC PERFORATION
4	1	32	Female	Shock*	328	27	8	Not related	Not related	SHOCK OF UNKNOWN ORIGIN
5	1	42	Male	Abdominal strangulated hernia	66	24	n/a	Not related	Not related	STRANGULATED SCROTAL INGUINAL HERNIA WITH BOWEL OBSTRUCTION
	2	42	Male	Haemorrhagic Shock	66	24	n/a	Not related	Not related	DIGESTIVE HEMORRHAGE WITH HEMORRAGIC SHOCK
6	1	43	Male	Craniocerebral injury	266	14	n/a	Not related	Not related	CRANIOCEREBRAL INJURY
Baby born to a vaccinated woman aged 20 years old				Exomphalos†	41	No dose 2	44	Improbable		EVISCERATION ON EXOMPHALOS

* Death of a vaccinated women during pregnancy at 8 weeks gestation

† Death in a baby born to a vaccinated mother aged 20 years, where the baby experienced a congenital anomaly of exomphalos after a full-term gestation

N/A = Not applicable

Supplementary table S5. Timing of exposure to each vaccine during pregnancy.

Total women reporting a pregnancy, N		1221
Total pregnancies reported, N		1238
Total pregnancies with known exposure (known date LMP), N		1037
Timing of conception and vaccination:		
Pregnancies ongoing at dose 1, n (%)		731 (70.5)
Conception 1-30 days post dose 1, up to 30 days pre-dose 2, n (%)		3 (0.3)
Conception 1-30 days post dose 1, >30 days pre-dose 2, N		48 (4.6)
Conception 1-30 days post dose 1, no dose 2 or birth outcome before dose 2, N		11 (1.1)
Conception >30 days post dose 1, <30 days pre-dose 2, N		42 (4.1)
Conception >30 days post dose 1, >30 days pre-dose 2, N		165 (15.9)
Conception >30 days post dose 1, no dose 2 or birth outcome before dose 2, N		21 (2.0)
Conception 1-30 days post 2, N		16 (1.5)
Exposure summary based on timing of conception and vaccination:		
Exposed to dose 1 only		487 (47.0)
Exposed to dose 2 only		258 (24.9)
Exposed to dose 1 and dose 2		244 (23.5)
Exposure to neither dose: conception post dose 1		32 (3.1)
Exposure to neither dose: conception post dose 2		16 (1.5)
Trimester at dose 1 (N=731) *, n (%)	1: <13 weeks	178 (24.4)
	2: 13-26 weeks	255 (34.9)
	3: 27-43 weeks	297 (40.6)
	Gestational age >43 weeks†	1 (0.1)
Trimester at dose 2 (N=502) *, n (%)	1: <13 weeks	142 (28.3)
	2: 13-26 weeks	176 (35.1)
	3: 27-43 weeks	184 (36.7)
Timing of exposure for all pregnancies with known exposure status:		
Dose 1, Trimester 1, Dose 2, Trimester 1		20
Dose 1, Trimester 1, Dose 2, Trimester 2		57
Dose 1, Trimester 1, Dose 2, Trimester 3		8
Dose 1, Trimester 1, no Dose 2 or pregnancy ended before dose 2		93
Dose 1, Trimester 2, Dose 2, Trimester 2		48
Dose 1, Trimester 2, Dose 2, Trimester 3		68
Dose 1, Trimester 2, no Dose 2 or pregnancy ended before dose 2		139
Dose 1, Trimester 3, Dose 2, Trimester 3		43
Dose 1, Trimester 3, no Dose 2 or pregnancy ended before dose 2		254
Conception post dose 1, Dose 2, Trimester 1		122
Conception post dose 1, Dose 2, Trimester 2		71
Conception post dose 1, Dose 2, Trimester 3		65
Exposed to neither dose: conception post dose 1		32
Exposed to neither dose: conception post dose 2		16
Gestational age >43 weeks†		1
Pregnancies with only dose 1 up to 90 days before conception, n		147

Pregnancies with only dose 2 up to 90 days before conception, n	16
Pregnancies with both doses 1 and 2 up to 90 days before conception, n	6
Women breastfeeding at the time of dose 1, n	1040
Women breastfeeding at the time of dose 2, n	920

Summary data are presented as number of pregnancies as some women reported more than one pregnancy during the study.

* Gestational age in weeks at the time of vaccination is calculated from the number of days between the date of each dose and date of LMP, divided by 7, rounded down to the nearest integer value, where date of LMP is known.

† Date of last menstrual period missing resulting in implausible timing for either dose 1 or dose 2

Supplementary table S6. Serious adverse events in women ever reporting a pregnancy: by exposure to each dose and overall.

	Exposed to dose 1 only during pregnancy	Exposed to dose 2 only	Exposed to both doses	All pregnant women, regardless of time of notification of pregnancy or exposure
Received dose 1, N	487	257	244	1221
SAE during 15 min of dose 1, n (%)	0 (0)	0 (0)	0 (0)	0 (0)
SAE >15 min-7 days post dose 1, n (%)	13 (2.7)	2 (0.8)	3 (1.2)	20 (1.6)
SAE 8-21 days post dose 1, n (%)	27 (5.5)	0 (0)	1 (0.4)	32 (2.6)
SAE 22-28 days post dose 1, n (%)	6 (1.2)	0 (0)	1 (0.4)	10 (0.8)
SAE >28 days post dose 1, before dose 2, n (%)	73 (15.0)	5 (1.9)	3 (1.2)	94 (7.7)
SAE 29-112 days post dose 1, no dose 2 †, n (%)	21 (4.3)	0 (0)	0 (0)	25 (2.0)
SAE 112 days post dose 1 to pregnancy outcome, no dose 2 †, n (%)	13 (2.7)	0 (0))	0 (0)	28 (2.3)
			0 (0)	
Received dose 2, N	315	257	244	988
SAE during 15 min post dose 2, n (%)	0 (0)	0 (0)	0 (0)	0 (0)
SAE >15mins-7 days post dose 2, n (%)	0 (0)	4 (1.6)	5 (2.0)	11 (1.1)
SAE 8-28 days (1m) post dose 2, n (%)	0 (0)	10 (4.1)	14 (5.7)	26 (2.6)
SAE 29-56 days (2m) post dose 2, n (%)	0 (0)	12 (4.9)	11 (4.5)	29 (2.9)
SAE 57-84 days (3m) post dose 2, n (%)	0 (0)	9 (3.7)	9 (3.7)	23 (2.3)
SAE 85-168 days (6m) post dose 2, n (%)	0 (0)	10 (4.1)	20 (8.2)	43 (4.4)
SAE 169 days post dose 2, to pregnancy outcome, n (%)	2 (0.6)	28 (11.5)	7 (2.9)	47 (4.8)
SAE any time after dose 1, n (%)*†	150 (30.8)	75 (29.2)	69 (28.3)	371 (30.4)
Total number of SAE†	168	86	76	413
Number of SAE per individual, median (range)	0 (0-3)	0 (0-3)	0 (0-3)	0 (0-3)

* Denominator is the number receiving dose 1.

†the cut-off date for analysing SAE data in pregnant women is the later date out of; pregnancy outcome, or, 112 days post dose 1 if delivery has already occurred

For the total number of SAE: 338 had 1 SAE, 27 had 2 SAEs, 7 had 3 SAEs, for a total of 413 (338+54+21)

Supplementary table S7: Incidence of reasons given for caesarean section.

Number of pregnancies delivered by caesarean section:	260
Number of reasons given for caesarean:	
1, n (%)	125 (48.1)
2, n (%)	99 (38.1)
3, n (%)	19 (7.3)
No reason given, n (%)	17 (6.5)
Frequency of each reason amongst all reported, n (%):	
Abnormal Labour	9 (3.5)
Abnormal Presentation	10 (3.8)
Cephalo-Pelvic Disproportion	55 (21.2)
Cervical Dystocia	8 (3.1)
Exceeded Term	2 (0.8)
Face Presentation	1 (0.4)
Failure Of Labour Induction	1 (0.4)
False Labour	2 (0.8)
Foetal Death	1 (0.4)
Foetal Distress	39 (15.0)
Foetal Dystocia	11 (4.2)
Grand Multiparity	2 (0.8)
History Of Uterine Rupture	2 (0.8)
Hyperkinesia	2 (0.8)
Hypokinesia	2 (0.8)
Placenta Praevia	13 (5.0)
Pre-Eclampsia	4 (1.5)
Precious Pregnancy	3 (1.2)
Premature Delivery	1 (0.4)
Premature Membranes Rupture	3 (1.2)
Premature Separation of Placenta	2 (0.8)
Prolonged Labour	2 (0.8)
Prolonged Pregnancy	4 (1.5)
Scheduled Caesarean	1 (0.4)
Sero Sanguineous Lump	1 (0.4)
Severe Anaemia	1 (0.4)
Short Interpregnancy Interval	13 (5.0)
Third Trimester Haemorrhage	1 (0.4)
Threatened Preterm Delivery	1 (0.4)
Tubal Ligation	7 (2.7)
Twin Pregnancy	6 (2.3)
Umbilical Cord Around Neck	10 (3.8)
Umbilical Cord Prolapse	1 (0.4)
Uterine Atony	8 (3.1)
Uterine Hypokinesia	2 (0.8)
Uterine Rupture/Pre-Rupture/Dehiscence	6 (2.3)
Uterine Scar	139 (53.5)
Water Membranes Rupture	3 (1.2)

Supplementary table S8: Pregnancy outcomes overall, by pregnancy subsets and by exposure during pregnancy

	Dose 1 subset	Pregnant within 30 days subset	Exposed to dose 1 only during pregnancy	Exposed to dose 2 only during pregnancy	Exposed to both doses during pregnancy	All pregnancies
**Number of pregnancies**	272	88	487	258	244	1238
No pregnancy outcome*	12 (4.4)	7 (8.0)	21 (4.3)	15 (5.8)	0 (0)	69 (5.6)
Number of pregnancies with outcome data	260 (95.6)	81 (92.0)	466 (95.7)	243 (94.2)	244 (100.0)	1169 (94.4)
**Pregnancies with an outcome:**	260	81	466	243	244	1169
Twin pregnancy	7 (2.7)	0 (0)	5 (1.1)	2 (0.8)	5 (2.0)	17 (1.5)
Triplet pregnancy	0 (0)	0 (0)	0 (0)	0 (0)	0 (0)	0 (0)
Miscarriage	8 (3.1)	10 (12.3)	21 (4.5)	15 (6.2)	2 (0.8)	55 (4.7)
Still birth†	4 (1.5)	0 (0)	19 (4.1)	4 (1.6)	5 (2.0)	31 (2.7)
Live birth†	248 (95.4)	71 (87.7)	427 (91.6)	224 (92.2)	237 (97.1)	1084 (92.7)
**Pregnancies with a still and, or live birth outcome:**	252	71	446	228	242	1114
Vaginal delivery‡	197 (78.2)	47 (66.2)	342 (76.7)	181 (79.4)	189 (78.1)	855 (76.6)
Caesarean delivery‡	56 (22.2)	24 (33.8)	103 (23.1)	47 (20.6)	54 (22.3)	260 (23.3)
**Number of babies born alive§:**	255	71	431	226	242	1100
Neonatal death: up to day 7	2 (0.8)	0 (0)	6 (1.4)	0 (0)	3 (1.2)	11 (1.0)
Neonatal death: day 8-28	0 (0)	0 (0)	0 (0)	0 (0)	0 (0)	0 (0)
Premature**	40/198 (20.2)	20/64 (31.3)	88/410 (21.5)	47/214 (22.0)	40/232 (17.2)	188/891 (21.1)
Congenital anomaly††	3 (1.2)	0 (0)	3 (0.7)	0 (0)	2 (0.8)	5 (0.5)
Neonatal problem	0 (0)	0 (0)	1 (0.2)	0 (0)	1 (0.4)	2 (0.2)
Prolonged hospitalisation	0 (0)	0 (0)	0 (0)	0 (0)	0 (0)	0 (0)
Low birth weight (all)‡‡	15/234 (6.4)	7/67 (10.4)	29/403 (7.2)	18/210 (8.6)	17/234 (7.3)	79/1032 (7.7)
Low birth weight (born ≥37 weeks gestation) ‡‡	8/149 (5.4)	2/42 (4.8)	13/305 (4.3)	9/159 (5.7)	10/186 (5.4)	33/672 (4.9)
Any neonatal adverse event§§	55 (21.6)	22 (31.0)	109 (25.3)	56 (24.8)	54 (22.3)	242 (22.0)

Data are n (%).

*Of pregnancies without an outcome, 55 pregnancies were lost to follow-up, 13 were denied later and 1 pregnancy outcome was not captured due to death of the mother

(ID: 1500791) at 8 weeks gestation

†Number of still births, live births and miscarriages add to one more than the number of outcomes available because one twin pregnancy resulted in a still and live birth.

‡Number of delivery types add to one more than the number of pregnancy outcomes available because one twin pregnancy resulted in a vaginal and caesarean delivery.

§Number of babies born alive equals number of live birth outcomes plus the number of twin pregnancies minus one due to the still birth in one set of twins.

**Baby born at less than 37 weeks gestational age (ICD 10: P073). Gestational age is based date of last menstrual period if available.

††Congenital anomalies are listed separately.

‡‡Birth weight <2.5kg. Data shown for non-missing data for birth weight and gestational age

§§Any of; low birth weight, premature, neonatal death, miscarriage, stillbirth, congenital anomaly.

Supplementary Table S9: Congenital anomaly SAE reports

	Days since dose 1	Days since dose 2	Gestational age (weeks) at onset*	MedDRA preferred term	Causality: dose 1	Causality: dose 2
1	247	No dose 2	SAE onset post birth	Inguinal hernia	Not related	
2	50	Birth before dose 2	45	Congenital tongue anomaly	Not related	
3	96	6	44	Umbilical hernia	Not related	Not related
4	114	52	37	Cleft lip	Not related	Not related
5	41	Birth before dose 2	44	Exomphalos‡	Improbable	

Gestational age calculated from date last menstrual period

* calculated from date of last menstrual period provided

‡ Baby died within a day of birth and is also included in the list of fatal SAEs.

2. SUPPLEMENTARY INFORMATION

Supplementary Information S1: Statistical methods

The number (%) of participants who received dose 2 was summarised overall and by timing of receipt of dose 2 in relation to dose 1 (i.e., within the original 56 days window (-14, +28 days) or outside that window or who did not receive dose 2).

No hypothesis testing of safety data was planned. Safety data were analysed descriptively. The number (n) and percent (%) of vaccinated individuals reporting a SAE, and number (n) and percent (%) of vaccinated individuals reporting a serious adverse reaction (SAR, considered related to the vaccine), were summarised for each safety subset (adult, child, and pregnancy), women in each overall exposure category (exposed to one or both vaccines during pregnancy), all women reporting a pregnancy at any time in the study and all non-pregnant individuals (i.e. those never reporting a pregnancy during the study). A pregnancy was considered exposed to a vaccine dose if the date of conception was prior to the vaccination, where date of conception was calculated as the date of last menstrual period (LMP) plus 14 days. Categorisation of exposure for this study was only possible for pregnancies for which a LMP date was available. SAEs were coded using the Medical Dictionary for Regulatory Activities (MedDRA 23.1). Incidence of SAEs were summarised overall and within the above groupings according to MedDRA preferred terms. Data were reported overall and by subset and exposure to vaccine categories during pregnancy. No comparisons were made across groups as these groups are sub-groups of the overall dataset and may overlap.

Summaries of the number and percentage of individuals never reporting a pregnancy who experienced a SAE are reported for different time periods: < 15 minutes of vaccination with dose 1; >15 minutes and up to 28 days post dose 1; > 28 days post dose 1 up to dose 2 or, if dose 2 was not received then up to 112 days post dose 1 (56 + 28 + 28 days); within 15 minutes of vaccination with dose 2; >15 minutes and up to one month post dose 2; anytime post dose 1 in all vaccinated with dose 1, up to one month post dose 2, or up to 112 days post dose 1 if dose 2 not received (56 + 28 + 28 days).

The number (%) of SAEs in individuals reporting a pregnancy at any time during the study were calculated for the time periods following vaccination; with 15 minutes of dose 1, >15 minutes to seven days post dose 1, 8-21 days post dose 1, 22-28 days post dose 1 and to >28 days post dose 1 up to dose 2, or if dose 2 not received; >28 days post dose 1 up to 112 days post dose 1 or pregnancy outcome whichever was later; <15 minutes of vaccination up to one month post dose 2 and >1 month post dose 2 or pregnancy outcome, whichever was later.

The pregnancy cohort was described with respect to exposure to each vaccine during pregnancy and the estimated trimester of pregnancy at the time of vaccination if LMP date were available. Trimesters were defined as trimester 1 (<13 weeks gestation), trimester 2 (13-26 weeks) and trimester 3 (27-43 weeks); exposure to each dose was defined as date of conception prior to the date of vaccination of each dose. The number (n) and percent (%) of women who reported current breastfeeding at the time of each dose was also reported.

Pregnancy outcomes were summarised as number (%) of each outcome out of all pregnancies overall, by pregnancy subsets and by exposure to vaccine during pregnancy. No comparisons are made across groups as these were sub-groups of the overall dataset and did not represent independent comparable groups for analysis. Outcomes of interest were miscarriage, stillbirth or live birth, vaginal delivery or caesarean section, twin pregnancies, and the rate of and reasons for caesarean sections. Congenital anomalies in babies were classified as SAEs. Among babies, data were recorded for number (%) of preterm livebirths and babies with low birthweight, with a congenital anomaly and who experienced neonatal death (within seven days and within 28 days). A summary of clinical observations is provided for the babies born to vaccinated mothers included in the infant subset.

The sample size assumptions for the study have been previously published [2]. The study originally planned to vaccinate approximately 500,000 people with Ad26.ZEBOV and MVA-BN-Filo in communities neighbouring areas with ongoing EVD transmission in order to achieve the sample size (110 confirmed EVD

cases among a population eligible to receive the study vaccines) required for primary evaluation of vaccine effectiveness This sample size for the original primary effectiveness evaluation was selected to provide 80% power with alpha = 0.05 for a two-sided test to demonstrate 70% vaccine efficacy (VE) under assumptions for coverage (percentage of vaccinated test negative individuals) ranging from 20-70% and ratio of test positives to test negatives ranging from 1:1 to 1:4 [2]. This sample size would provide 80% power to demonstrate 50% vaccine efficacy (VE) under assumptions for coverage ranging from 50-70% and ratio of test positives to test negatives ranging from 1:2 to 1:4.

The subsets of individuals with active follow-up for safety reporting allowed for 95% probability to detect at least one rare event SAE; if the true incidence of that SAE was 1.2% with 250 individuals or was 0.6% with 500 individuals or was 0.3% with 1000 individuals i.e. with 500 individuals, there is a 95% probability of observing one event if the true incidence of that event is 0.6%.

References

(See main manuscript)

[2] Watson-Jones D, Kavunga-Membo H, Grais RF, Ahuka S, Roberts N, Edmunds WJ, et al. Protocol for a phase 3 trial to evaluate the effectiveness and safety of a heterologous, two-dose vaccine for Ebola virus disease in the Democratic Republic of the Congo. BMJ Open 2022;12:e055596. https://doi.org/10.1136/bmjopen-2021-055596.
